# Further observations on a principal components analysis of head-related transfer functions

**DOI:** 10.1038/s41598-019-43967-0

**Published:** 2019-05-16

**Authors:** Parham Mokhtari, Hiroaki Kato, Hironori Takemoto, Ryouichi Nishimura, Seigo Enomoto, Seiji Adachi, Tatsuya Kitamura

**Affiliations:** 1 Center for Information and Neural Networks (CiNet), National Institute of Information and Communications Technology (NICT), Seika-cho, Kyoto Japan; 2 Advanced Speech Translation Research and Development Promotion Center, National Institute of Information and Communications Technology (NICT), Seika-cho, Kyoto Japan; 3 Chiba Institute of Technology, Narashino Chiba, Japan; 4 Resilient ICT Research Center, National Institute of Information and Communications Technology (NICT), Sendai, Miyagi Japan; 5 Fraunhofer Institute for Building Physics, Stuttgart, Germany; 6 Konan University, Kobe Hyogo, Japan; 7 Present Address: Toyama Prefectural University, Imizu, Toyama, Japan

**Keywords:** Physics, Engineering, Psychology

## Abstract

Humans can externalise and localise sound-sources in three-dimensional (3D) space because approaching sound waves interact with the head and external ears, adding auditory cues by (de-)emphasising the level in different frequency bands depending on the direction of arrival. While virtual audio systems reproduce these acoustic filtering effects with signal processing, huge memory-storage capacity would be needed to cater for many listeners because the filters are as unique as the shape of each person’s head and ears. Here we use a combination of physiological imaging and acoustic simulation methods to confirm and extend previous studies that represented these filters by a linear combination of a small number of *eigenmodes*. Based on previous psychoacoustic results we infer that more than 10, and as many as 24, eigenmodes would be needed in a virtual audio system suitable for many listeners. Furthermore, the frequency profiles of the top five eigenmodes are robust across different populations and experimental methods, and the top three eigenmodes encode familiar 3D spatial contrasts: along the left-right, top-down, and a tilted front-back axis, respectively. These findings have implications for virtual 3D-audio systems, especially those requiring high energy-efficiency and low memory-usage such as on personal mobile devices.

## Introduction

Humans localise sound-sources by using binaural and monaural acoustic cues contained in head-related transfer functions (HRTFs), which describe the direction-dependent filtering due to sound-wave interactions with the head and pinnae (outer ears). However, HRTFs are unique to each individual because each person has a unique head and pinna shape. For spatial audio systems aiming to serve a large population of listeners, this implicates potentially huge requirements for memory (or storage) space. To help resolve this issue, previous studies proposed dimensionality reduction to a small number of eigenmodes by principal components analysis (PCA) of HRTFs measured on groups of individuals^1,2^, and even further reduction was achieved by vector quantization of the PC weights^3^.

However, it is still not established how many eigenmodes are needed to ensure sufficient accuracy in either HRTF representation or sound-localization performance. Although it was previously suggested that five eigenmodes might be sufficient^1^, this still led to localization errors especially in the judgment of the up-down angle of presented stimuli^1^. On the other hand, a psychoacoustic study^4^ that progressively smoothed HRTFs with a diminishing number of discrete cosine transform (DCT) coefficients, concluded that more than 16, and as many as 32 DCT coefficients are required for listeners to be unable to distinguish between virtual and real sound-sources. Their conclusion that the fine structure of HRTFs is not important for spatial hearing^4^ accords with the earlier observations on HRTFs reconstructed from a limited number of eigenmodes^1^, but a direct comparison between the two representation methods (PCA vs DCT) has not yet been made.

Additionally, there remain questions concerning both the generality and the spatial interpretability of HRTF eigenmodes. Usually, eigenvectors yielded by PCA are sensitive to the exact composition of the data being analyzed. Therefore, if similar eigenvectors are obtained across two or more, independently sampled datasets, it can be taken to indicate a robust, underlying relationship among the measured variables, especially if the datasets were obtained by different experimental methods or on disjoint sample populations. Such was the case when the top five eigenmodes of HRTFs measured in two different laboratories were reported to be highly correlated^2^, despite methodological differences and, more importantly, different groups of individuals. However, to the best of our knowledge, no subsequent study has yet confirmed or extended that comparison.

Concerning spatial interpretability, the first eigenmode has been shown to represent HRTF variation along the left-right axis (i.e., ipsilateral vs contralateral)^1^. Perhaps unsurprisingly, when the data subjected to PCA were restricted to the median plane and thus varied only in elevation (or polar) angle, two studies reported^5,6^ that the first two eigenmodes represented variations mainly in elevation (low vs high) and along the front-back axis, respectively. However, this has not yet been confirmed for HRTF datasets that include a wide range of source locations around a listener.

While all the studies cited thus far used HRTFs obtained by acoustic measurements, calculation of HRTFs by numerical simulation — i.e., head and pinna imaging followed by computer simulation of acoustic wave propagation and scattering^7–11^ — offers several advantages. First, on an individual basis, simulation methods eliminate variability due to involuntary movements of the head or other parts of the body, or incidental movements of the microphones at the listener’s ears, during measurement. Second, on an inter-individual basis, variabilities due to the initial alignment of the listener’s head and torso, and the precise position of the microphones in the ear-canals, can be eliminated. Third, simulation methods can overcome practical measurement artefacts caused by the finite (non-point) size of sound-source (loudspeaker) and observation (microphone) transducers, acoustical interference of nearby objects (e.g., speaker stand, listener’s chair, head-rest, microphone cables), and changes in the acoustic characteristics of loudspeakers and microphones over time. Elimination of such intra- and inter-individual variabilities and measurement artefacts can help secure high reliability for the results of the HRTF analyses reported here.

The present study therefore used acoustic simulation methods, to investigate the following three questions: (i) how many eigenmodes are needed to achieve the auditory-perceptual fidelity — i.e., indistinguishability of virtual and real sound-sources — previously reported^4^ for DCT basis functions; (ii) on the question of generality, how similar are the eigenmodes to those reported previously^1,2^ based on acoustic measurements; and (iii) do the eigenmodes have clear spatial interpretations. These questions are addressed with the help of head and pinna geometries of 19 adults measured by magnetic resonance imaging (MRI); followed by finite-difference time domain (FDTD) simulation to calculate HRTFs of all 38 ears, at each of 1250 surrounding spatial locations a distance 1 m from the head center. After mean subtraction per pinna to retain directional transfer functions^1,2^, the entire set of 47,500 HRTFs was submitted to PCA. The same dataset was then represented using DCT coefficients^4^, and the representation accuracies were compared with those of eigenmode analysis in order to infer a lower bound on the required number of eigenmodes.

## Results and Discussion

### Required number of eigenmodes

To infer a lower bound on the number of eigenmodes necessary for auditory-perceptual sufficiency, we related our eigenmode analysis to previous psychoacoustic results based on a reconstruction of HRTFs with {8, 16, 32, …} DCT coefficients^4^. In particular, we reconstructed all 47,500 HRTFs first with an increasing number of eigenmodes^1^, then again with an increasing number of DCT coefficients^4^. To relate the two sets of results, the effect in each case was quantified by a commonly used spectral representation error (the root mean square difference between original and reconstructed HRTFs in dB) within the range 0.5–14 kHz.

**Figure 1:**
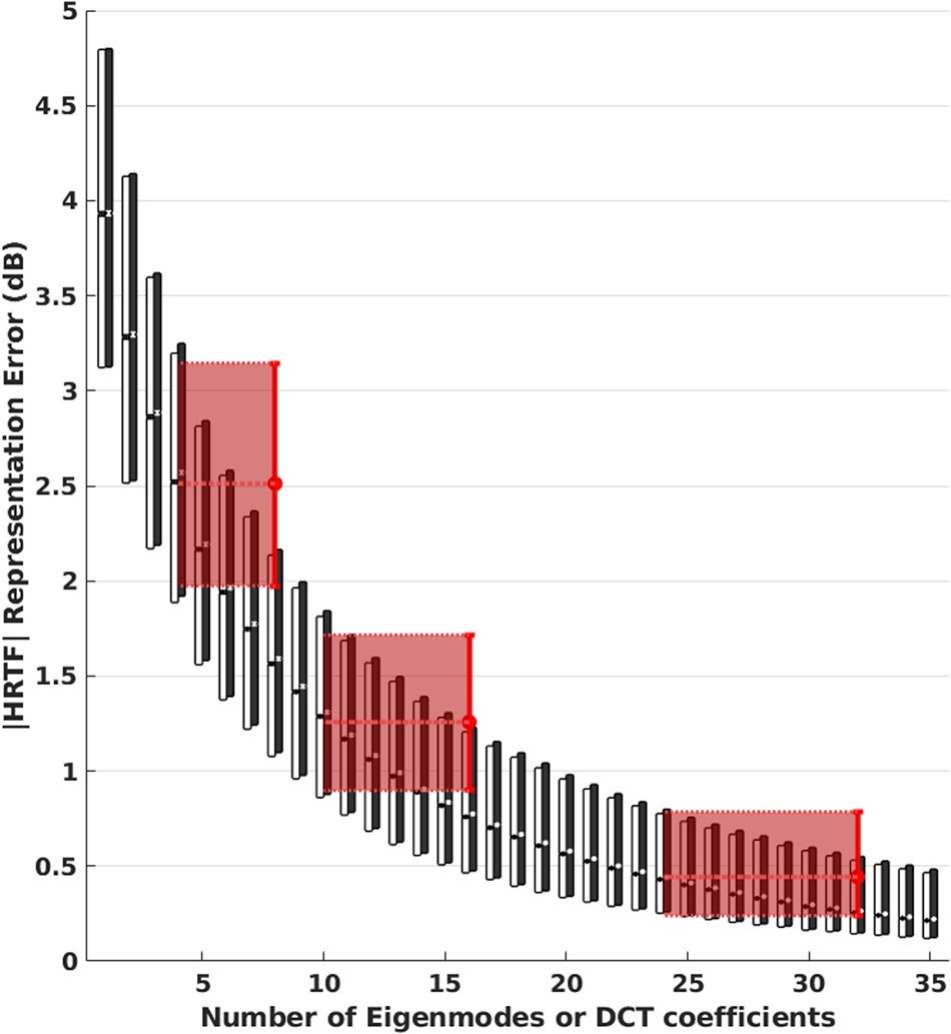
Correspondence between PCA and DCT representations. Each error distribution (*n* = 47,500) is shown by its median and 1^st^ & 3^rd^ quartiles. White boxes: all-inclusive PCA. Black boxes: cross-validation PCA (holding out one individual at a time). Red vertical thick lines: DCT with 8, 16, and 32 coefficients. Pale red dotted lines and patches: horizontal extension of DCT results to closest-matching PCA error distributions (i.e., to 4, 10, and 24 eigenmodes respectively).

As shown in Fig. 1, representing HRTFs with empirical eigenmodes incurred lower errors than with the same number of DCT coefficients, although the gap diminished with increasing number of coefficients. This can be explained by the fact that the DCT’s cosine basis functions are generic and treat the entire available frequency range equally, while the eigenmodes derived by PCA are adapted to the data and therefore place greater modelling accuracy on those frequency regions that require it. Despite this inherent difference, it is still reasonable to directly compare the two methods and determine to what extent PCA offers a greater reduction in dimensionality.

The black boxes in Fig. 1 indicate that the same observation holds in the case of leave-one-person-out cross-validation — i.e., when the eigenmodes used to reconstruct each HRTF were derived only after holding out all of that person’s data. Compared with all-inclusive analysis, cross-validation increased the first quartile, median, and third quartile of representation errors only by as much as 0.01 dB, 0.02 dB, and 0.08 dB, respectively. These results underscore the robustness of the eigenmodes in the face of individual differences.

To find a correspondence between the two methods, in Fig. 1 the median and quartiles of the DCT-representation errors were horizontally extended to the closest-matching statistics of PCA-representation errors. As a result, we found that 8, 16, and 32 DCT coefficients approximately corresponded to 4, 10, and 24 eigenmodes respectively. Here, for completeness we included the results at 8 DCT coefficients (corresponding to 4 eigenmodes), even though at that level of HRTF smoothing listeners were found to distinguish between real and virtual sound-sources^4^. On the basis of previous psychoacoustic results^4^ we infer that more than 10, and as many as 24 eigenmodes may be necessary for listeners to be unable to distinguish between virtual and real sound-sources. This range may be further narrowed down in the future by conducting auditory discrimination experiments as in the previous study^4^, to find the threshold (between 10 and 24 eigenmodes) at which listeners can no longer distinguish between real and virtual sound-sources beyond chance level.

Also in Fig. 1, as the number of eigenmodes increases, the median error tends to move away from the centre of the distribution and towards the first quartile. This may be explained simply as a flooring effect, as the representation error is bounded below by zero.

### Generality of eigenmodes

**Figure 2:**
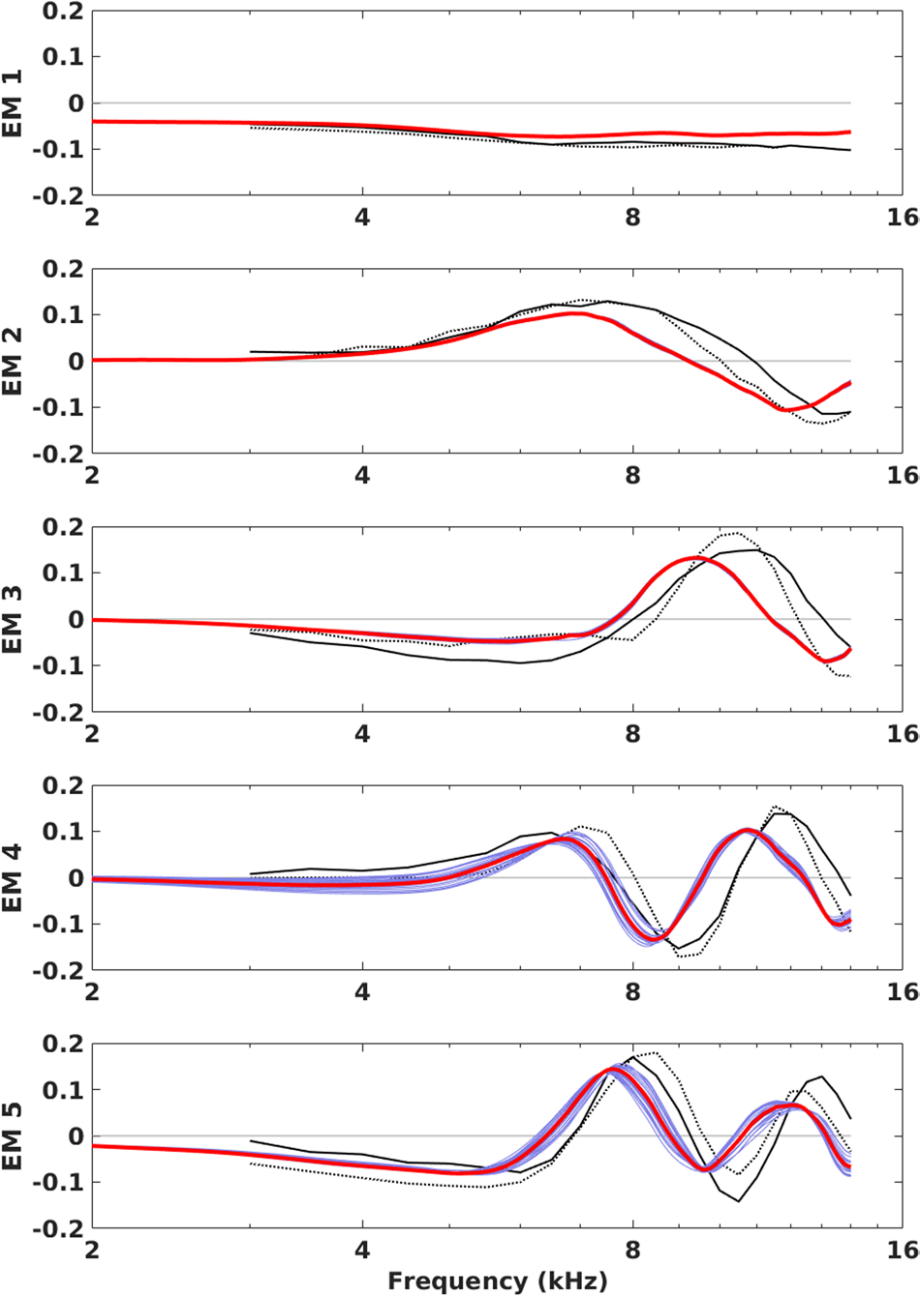
Top five eigenmodes of human HRTFs. Data from two previous studies shown in black dotted^1^ and solid^2^ lines (reproduced from Fig. 1 of ref.^2^). Our data shown in red (all-inclusive analysis) and pale blue (19 separate analyses leaving out one person at a time).

To answer the second question posed in the Introduction, we compare our PCA results with the top five eigenmodes reported previously. Similar to those studies^1,2^, the top five principal components of our simulated HRTFs together accounted for 89.0% of the total variance (previously reported as 90.3%^1^ and 89.5%^2^). More remarkably as shown in Fig. 2, the top five eigenmodes were similar in shape to those reported previously, with only minor differences including a visible but small shift downwards in frequency.

As listed in Table 1, before any frequency scaling the correlation coefficients between our top five eigenmodes and those from the two earlier studies ranged from 0.57 to 0.94, with mean values of 0.80 and 0.76 respectively with each study. After optimal scaling in frequency^12^ (i.e., shifting of all five eigenmodes along the logarithmic frequency axis to maximise the mean correlation) the correlations ranged from 0.88 to 0.99, with mean values of 0.98 and 0.94 respectively. These results confirm quantitatively that the eigenmode shapes are similar to those reported previously, despite the substantial differences in the methods used to obtain the HRTFs (e.g., simulation vs measurement, blocked vs open ear-canal, exclusion vs inclusion of torso and other bodily reflections) and equally importantly, a distinct group of individuals.

**Table 1:** Eigenmode correlations with previous studies.

Eigenmode	K&W		M&G	
	(a)	(b)	(a)	(b)
1	0.93	0.97	0.91	0.96
2	0.94	0.99	0.86	0.97
3	0.79	0.98	0.72	0.95
4	0.57	0.99	0.62	0.88
5	0.78	0.99	0.70	0.93
mean	0.80	0.98	0.76	0.94

Correlation coefficients between the top five eigenmodes and those of two previous studies (K&W^1^, M&G^2^). (a) before any frequency scaling. (b) after optimal frequency scaling^12^ (+8.6%^1^ and +7.8%^2^) to maximise the mean correlation.

The optimum frequency scale-factors needed to best align the red lines in Fig. 2 with the black dotted and solid lines, respectively, were +8.6% and +7.8%. Although a direct physiological comparison is not possible, these numbers suggest that as a group, the acoustically-relevant head and pinna dimensions of our 19 adults (5 women, 14 men) were proportionally larger than those of the 10 young adults (7 women, 3 men)^1^ and 8 subjects (3 women, 5 men)^2^ who participated in the previous studies.

To rule out the possibility that these group differences were skewed by any one individual, Fig. 2 also shows the results of PCA on a leave-one-person-out basis. While the fourth and fifth eigenmodes display larger variability than the first three (indeed the pale blue lines are all but hidden behind the red lines in the first 2–3 eigenmodes), all five eigenmodes appear remarkably stable across the 19 separate analyses.

### Spatial interpretation of eigenmodes

Given that these eigenmodes underlie HRTF variations across a wide range of sound-source directions, what spatial contrasts do they each encode?

**Figure 3:**
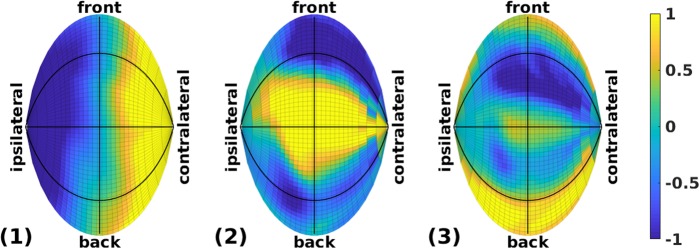
Spatial maps of top three eigenmodes. Hammer projection of mean weights on each eigenmode, at all 1250 farfield locations. Black reference lines indicate the median plane (vertical line), the frontal plane (horizontal line), and the horizontal plane (curved lines). The color scale extends to ±1 standard-deviation.

Our farfield spatial map in the first panel of Fig. 3 confirms^1^ that eigenmode 1 encodes the contrast between ipsilateral and contralateral sound sources (note that in the creation of these spatial maps, all right-pinna maps were reflected about the median plane so that left and right correspond to ipsilateral and contralateral, respectively). Referring to the shape of the first eigenmode in Fig. 2, the head shadow reduces the overall level received at the contralateral ear with a low-pass effect in frequency: progressively shorter wavelengths have a more pronounced shadow owing to lower levels of diffraction around the head.

Additionally, the second panel in Fig. 3 shows that eigenmode 2 encodes the contrast between high- and low-elevation sound sources. Referring to the shape of the second eigenmode in Fig. 2, HRTFs at higher elevations tend to emphasise a broad mid-frequency range (~4–9 kHz) and de-emphasise higher frequencies (~10–14 kHz), while the opposite occurs at lower elevations. This effect can be ascribed to the pinna: at increasingly higher elevations the spectral notches caused by the pinna typically shift upward in frequency^8,9^, leaving the mid-frequency range notch-free and therefore relatively raised in level.

Furthermore, the third panel in Fig. 3 shows that eigenmode 3 encodes mainly the contrast between upper-front and lower-back sound sources, thus forming a front-back axis tilted upward by about 30–40°. Referring to the shape of the third eigenmode in Fig. 2, HRTFs at lower-back directions tend to emphasise a mid- to high-frequency range (~8–11 kHz) and de-emphasise both lower (~3–7 kHz) and higher (~12–14 kHz) ranges, while the opposite occurs for sound sources at upper-front. This effect is also likely to be caused mainly by the pinna.

All three spatial maps in Fig. 3 appear slightly rotated clockwise about the vertical axis; this may be attributed to the pinna flare-angle^11^ (the laterally outward tilt of the pinna relative to a parasagittal plane), which in our group was 23° on average (range 17–35°). Similarly, the upward tilt of eigenmode 3 may partly be attributed to the pinna rotation-angle^11^ (i.e., the generally backward tilt of the pinna’s major axis relative to the vertical), which in our group was 13° on average (range 1–24°). Spatial maps for the fourth and higher eigenmodes were increasingly complex and difficult to interpret in simple terms.

These spatial interpretations are intuitively appealing, as they indicate that the top three eigenmodes, together accounting for 81.8% of the total variance in the HRTFs, are approximately congruent with the conventional orthogonal axes in three-dimensional (3D) space. Nevertheless, as inferred earlier, many more than three eigenmodes are needed to reconstruct the HRTFs of an individual pinna with sufficient accuracy for sound externalization and localization. It is also worth emphasising that even with sufficiently detailed and individualised HRTFs, manipulation of stimuli by these top three eigenmodes alone may not be enough to move a listener’s auditory image along the respective spatial axis; while eigenmode 1 does include inter-aural level differences to a large extent, to move an image would require co-variation of other cues such as the inter-aural time difference (for the left-right axis) and HRTF notches (for elevation angle). Adequate formation of narrow-bandwidth notches in particular would likely require co-variations in a sufficiently higher number of eigenmodes.

A limitation of the simulation approach used here, is that it relies on a sufficiently detailed and accurate measurement of an individual’s head and ear geometry. While the influence of imaging errors or artefacts is not readily known, a previous study^13^ on the effects of the precise position of the acoustic source at the pinna showed that HRTF variations were restricted to frequencies higher than about 12 kHz. Another more general limitation concerns the availability of measurement facilities (such as MRI) which are quite different from the facilities needed for acoustic measurements.

One future line of investigation would be to estimate the eigenmode weights from pinna anthropometric measurements that are readily accessible — similar to previous work that estimated the HRTF peaks corresponding to concha-depth^10^ and pinna-vertical^11^ resonances. This would allow to obtain individualised HRTFs while bypassing detailed pinna imaging and acoustic simulation. Our results here suggest that the weights on at least the first 10 eigenmodes would need to be estimated with sufficient accuracy, as a minimal requirement for HRTF personalisation. While estimation of various types of dimensionally reduced HRTFs from head and pinna anthropometry has been reported in several previous PCA-based studies e.g.^14–16^, none have yet attempted to use the more comprehensive list of pinna anatomical landmarks^10,11^, distances between which were shown to include acoustically highly relevant measures.

To conclude, our findings on the required number of HRTF eigenmodes, their spatial interpretation, and their generality across different methods and populations, have implications for virtual 3D audio systems, especially those that need to operate with high energy-efficiency and low memory-usage such as on personal mobile devices. Additionally, the ability of our results to both confirm and extend previous studies based on acoustic measurements, highlights the maturity and utility of numerical acoustic simulation with the FDTD method.

## Methods

### Head and pinna measurements

The head and pinna geometries used here were identical to the data described in previous publications^10,11^. Briefly, geometries of 19 adults were measured by MRI with either a Siemens 3.0 T Magnetom Trio (5 women and 13 men) or a Shimadzu-Marconi 1.5 T Magnex Eclipse (1 man) installed at the Brain Activity Imaging Center, ATR-Promotions Inc. (Kyoto, Japan). All participants gave written informed consent for the experimental procedures. This study was approved by, and all experiments were conducted in accordance with the relevant guidelines and regulations of, both the ATR Human Subject Review Committee and the NICT Committee on Bioinformation Research Ethics. Imaging data acquired at resolution 1.0 mm (11 participants) or 1.2 mm (8 participants) were processed with in-house software to binarize the grayscale (air vs non-air), form a contiguous head volume, block the ear canals at their entrance plane, and downsample to isovoxel resolution 2.0 mm for acoustic simulations.

### Acoustic simulation

The FDTD acoustic simulator was described and evaluated previously^8–10,17^. Briefly, the acoustic pressure and particle velocity were calculated in a leap-frog fashion in time and on a staggered 3D spatial grid encompassing the head down to the neck. Calculations were updated only at cells representing air (not inside the head volume); and every boundary between an air and a head cell was treated as having a frequency-independent, normal acoustic impedance^18^ equivalent to an air-water interface. The computation domain was surrounded by a 10-cell optimal perfectly matched layer^19^ to minimise artefactual reflections. To increase computational efficiency (both runtime and memory usage), the 3D grid was just large enough to enclose the head with about 3 cm clearance, and farfield pressure responses at 1250 spatial locations a distance 1 m from the head center were calculated by Kirchoff-Helmholtz integration^20^. The farfield locations complied with a head-centered interaural polar coordinate system^21^ (all combinations of 25 lateral angles and 50 polar angles). With a broadband acoustic source at one ear (directly adjacent to the center of the blocked ear-canal), responses were calculated on a 5 ms timeline at a sampling rate of about 1.3 MHz (for numerical stability and accuracy); these were later downsampled by a factor of ten, and normalised by the equivalent free-field responses (with the source at head-center) to obtain HRTFs. Ambient air temperature was set to 20 °C.

### PCA methods

Before PCA, all HRTFs were resampled by cubic spline interpolation at 50 Hz resolution from 500 Hz to 14 kHz; thus the input to PCA was a matrix of size 271 frequency bins ×47,500 HRTFs. Correlations between our eigenmodes and those of previous studies^1,2^ were calculated within the common frequency range 3–14 kHz.

### DCT methods

As in the previous study^4^, DCT coefficients were calculated on HRTFs in the frequency range 350 Hz to 15 kHz. While HRTFs smoothed by retaining a certain number of DCT coefficients were represented in the same frequency range, to be consistent with the PCA results HRTF representation errors were calculated within the slightly narrower range 500 Hz to 14 kHz.

## Data Availability

The HRTF data analysed during the current study are available from the corresponding author on reasonable request.
